# Detection of Gaps in Concrete–Metal Composite Structures Based on the Feature Extraction Method Using Piezoelectric Transducers

**DOI:** 10.3390/s19081769

**Published:** 2019-04-13

**Authors:** Paritosh Giri, Spandan Mishra, Simon Martin Clark, Bijan Samali

**Affiliations:** 1 Department of Physics and Astronomy, Macquarie University, North Ryde 2109, Australia; 2 Department of Industrial and Manufacturing Engineering, Florida A&M University—Florida State University College of Engineering, Tallahassee, FL 32310, USA; mishrsp@eng.fsu.edu; 3 Department of Earth and Planetary Sciences, Macquarie University, North Ryde 2109, Australia; simon.clark@mq.edu.au; 4 Centre for Infrastructure Engineering, School of Computing, Engineering and Mathematics, Western Sydney University, Penrith 2751, Australia; b.samali@westernsydney.edu.au

**Keywords:** concrete-filled steel tubes, piezoelectric transducers, structural health monitoring, feature extraction, partial least square regression, gaps, debonding

## Abstract

A feature extraction methodology based on lamb waves is developed for the non-invasive detection and prediction of the gap in concrete–metal composite structures, such as concrete-filled steel tubes. A popular feature extraction method, partial least squares regression, is utilised to predict the gaps. The data is collected using the piezoelectric transducers attached to the external surface of the metal of the composite structure. A piezoelectric actuator generates a sine burst signal, which propagates along the metal and is received by a piezoelectric sensor. The partial least squares regression is performed on the raw sensor signal to extract features and to determine the relationship between the signal and the gap size, which is then used to predict the gaps. The applicability of the developed system is tested on two concrete-metal composite specimens. The first specimen consisted of an aluminium plate and the second specimen consisted of a steel plate. This technique is able to detect and predict gaps as low as 0.1 mm. The results demonstrate the applicability of this technique for the gap and debonding detection in concrete-filled steel tubes, which are critical in determining the degree of composite action between concrete and metal.

## 1. Introduction

Concrete-metal composite structures, such as concrete-filled steel tubes (CFSTs), constitute critical components of civil infrastructure such as columns, piles, bridge piers, railway decks and roofs [1]. These composites have many advantages compared to columns exclusively made of steel or reinforced concrete [2,3]. The steel member of the composite structure provides high tensile strength and ductility, while the concrete member provides high compressive strength and stiffness. Due to this reason, CFSTs are abundantly used in building and bridge construction [4]. The interaction between the steel surface and concrete delays the local buckling of steel tube and the confinement from the metal increases the strength of concrete. This interaction also reduces the drying shrinkage and deterioration of concrete [5,6]. However, factors such as shrinkage of concrete, temperature variations and poor construction quality lead to debonding and gap formation between metal and concrete surface [7]. The gap due to the shrinkage of concrete is mostly observed in early age concrete due to the rapid shrinkage rate of restrained concrete inside the steel tube [8]. This gap reduces compressive and flexural behaviour of the composite structure and decreases the load carrying capacity and ductility [9]. Due to the confinement, such defects cannot be identified using a visual inspection technique and a remote sensing method is needed to detect and monitor any gaps.

Lamb wave techniques are conventionally used in different non-destructive testing applications [10,11,12,13]. In most applications, lamb wave is generated using an embedded or surface-mounted piezoelectric transducers. Piezoelectric transducers can act both as transmitter and receiver. When used as an actuator, a piezoelectric transducer generates a lamb wave due to the mechanical deformation when a voltage is applied between the electrodes of the piezoelectric material. Similarly, when used as a sensor, the mechanical energy is transformed into electrical energy [14]. Piezoelectric transducers operate either in a pitch-catch mode or pulse-echo mode. The pitch-catch mode is generally used to detect defects between the transducers. In this mode, the lamb wave signal generated by an actuator passes through the defect and reaches the sensor on the other side. The pulse-echo mode consists of transducers on the same side of the defect. The signal generated by the transducer is reflected by the defect that is received by the sensor. A single transducer can be used for actuation as well as sensing in a pulse-echo mode [15]. The lamb wave signal upon reaching the defect reflects the portion of the wave, which is proportional to the difference in the stiffness and density of the material [16]. The information on the extent of the defect and the location can be obtained by the detailed investigation of the reflected wave. The electromechanical impedance-based health monitoring technique was utilised to detect different damages in metal plates based on statistical metrics [17]. The debonding on hidden frame supported glass curtain wall was detected using nonlinear ultrasonic modulation method, where the feature components were extracted from the piezoelectric sensor data [18]. The spectral finite element method was developed to study the lamb wave propagation in the steel rebar and detect debonding defect between the concrete-steel interface in a reinforced concrete beam [19,20]. A wavelet packet-based structural damage index was utilised with the embedded piezoelectric sensor, called as smart aggregates in the concrete, to detect defects such as cracks and bond slips between concrete and steel [21,22,23]. Similarly, embedded piezoceramic-based smart aggregates were used to detect compactness of concrete and debonding in CFSTs using different algorithms, such as the time reversal method, wavelet packet analysis and the spectral element method [24,25,26,27]. A detailed study on the interfacial debonding detection, by considering concrete as a meso-scale structure for numerical simulations of stress wave propagation, was implemented in rectangular CFSTs using embedded piezoelectric sensors [28,29]. Surface-mounted and embedded piezoelectric patches and a circular CFST column was used to develop a coupling model, using the finite element numerical analysis technique for sensitivity analysis of the sensors and detection of interfacial debonding defects [30]. A debonding between ultra-high performance concrete and a steel plate was detected with piezoelectric sensors, using impedance analysis and the wave propagation technique [31]. Statistical damage indices were proposed based on guided lamb waves to detect gaps and debonding between the CFRP plate and concrete [32,33].

Various physical- and statistical-based techniques have also been used to extract damage sensitive features from lamb waves [34]. One common analysis technique is principal component analysis (PCA), which uses an orthogonal transformation to reduce large dimensional and correlated data into lower dimensional and linearly uncorrelated features [35]. An artificial fatigue crack propagation signal was generated and the PCA technique was applied, which differentiated the noise from the generated signal automatically [36]. The results demonstrate that the PCA technique can discriminate between different signal types. The nonlinear PCA technique was used, which projected the lamb wave signal data into curves beside the traditional orthogonal projection for structural assessment [37]. A minor PCA technique was used to separate the temperature dependent data from the damage data for accurate measurement of structural responses [38]. The presence of damage along with the type of damage and the extent of damage was determined using the lamb wave testing method coupled with PCA in composites [39]. In this study, the feature extracted from the PCA was used to train the pattern recognition algorithms, such as K-nearest neighbours and neural-network, to perform further classification. Mishra et al. [40] have used PCA-based analysis of a multivariate cumulative sum chart to monitor a structure undergoing fatigue loading. They found that features in the analysis were sensitive to minimal changes in the physical properties of the structure.

Mishra and Vanli [41] have used principal component regression (PCR) to predict delamination on a composite structure. The predicted delaminations were then used in a Weiner process-based degradation model to predict the remaining useful life of the structure. PCR is a two-step process that is based on the PCA technique. It is used to analyse multiple regression data by adding a degree of bias to the regression estimates. It helps to remove collinearity and eliminates unnecessary noise by removing the lesser principal components. However, these techniques do not consider the response variable at all. This leads to a risk that some useful information will end up in the discarded principal components and, as a result, some noise will end up in the regression model. An alternate approach is to use the partial least square regression (PLSR) technique, which considers the response variable. This technique does not impose restrictions employed by PCR technique and the models effectively fit the response variable with even fewer components. The advantage of principal component-based techniques for gap/debonding detection in concrete-metal composite structures has not been exploited as yet. This study aims to apply the PLSR technique on the lamb wave received by a piezoelectric transducer in a pitch-catch configuration, in order to predict the gap in concrete-metal composite structures like CFSTs. An initial investigation on gap detection in a concrete-metal composite structure, using statistical analysis techniques, has been carried out [42]. The piezoelectric transducers were attached to the external surface of the metal and the signal with no gap is considered as a reference signal at an initial state and the signals at different gap values are considered as a current state for gap prediction. The proposed technique is a fully non-invasive, economical and non-destructive approach to gap detection using a minimal number of transducers without the need for complex structural modelling.

The manuscript is organised as follows: Section 2 discusses the theory behind the partial least squared regression used for the feature extraction as well as damage prediction. Section 3 discusses the material used and the experimental procedure used to collect data. In Section 4, the results of the numerical analyses are given and finally, Section 5 concludes this research paper.

## 2. Partial Least Squared Regression Technique

Principle component analysis (PCA) is a popular multivariate statistical analysis method for dimensional reduction, which works by reducing the correlation between the variables. The transformed variable $z_{j}$ is a linear combination of the original variables $\left[x_{1},x_{2},\ldots x_{p}\right]$ and captures the maximum possible variance. If the original set of p variables are a linear combination of q new variables, then the first q principal components will be sufficient to capture all the variance and the remaining p−q principal components can be representative of noise present in the data [40]. Partial least squared regression (PLSR) combines the features of both generalized principal component analysis and multiple linear regression, whose primary objective is to find a set of independent variables using common structure between dependent and independent variables.

**Figure 1:**
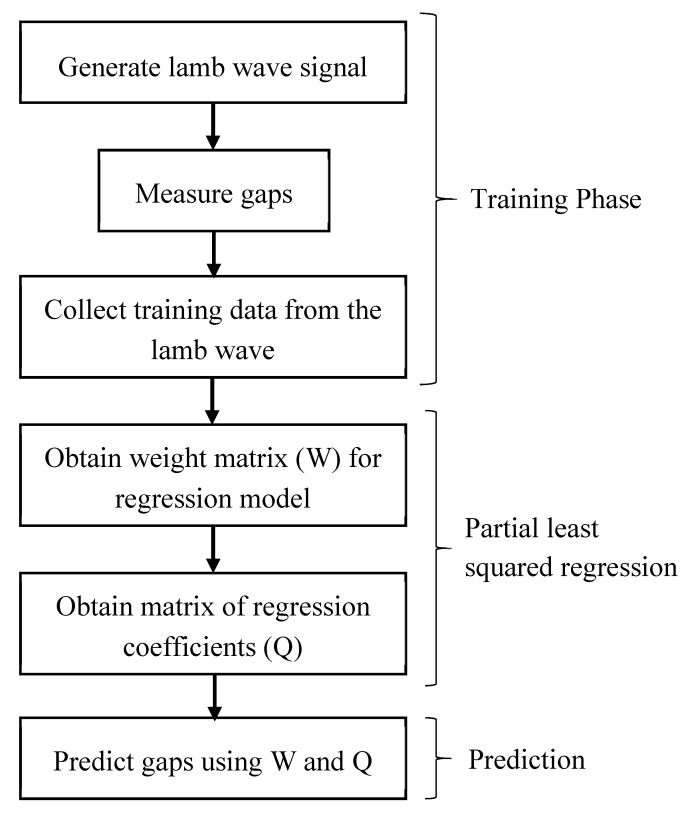
Flowchart to predict gaps using the training data from lamb waves.

The chief objective of PLSR is to build a linear model Y=XB+E, where Y is an n×m variables response matrix representing damage size (m=1, because damage size is a scalar quantity), X is an n×p variables predictor matrix representing lamb wave sensor signals, B is a p×m regression coefficient matrix, and E is a noise term for the model with a same dimensions of ***Y***. The variables in X and Y are centered. This is performed by subtracting the mean of the variables, which is then scaled by dividing with their standard deviations. Let us say one has dependent variable Y and many predictor variables X, some of which are highly correlated. A regression using factor extraction for this type of data computes the factor score matrix T=XW for an appropriate weight matrix ***W***, and then considers the linear regression model Y=TQ+E, where Q is a matrix of regression coefficients (loadings) for T, and E is an error term. Once the loadings Q are computed, the above regression model is equivalent to Y=XB+E, where B=WQ, which can be used as a predictive regression model. A p×c factor loading matrix P gives a factor model X=TP+F, where F is the unexplained part of ***X*** [43]. The loading parameters P, Q and regression coefficients B were determined using the NIPALS Algorithm [44]. The detailed flowchart on the use of PLSR for the prediction of gaps using the lamb waves is given in Figure 1.

It should be noted that it is possible to estimate as many partial least square components as the rank of original matrix X; however, not all the components are useful because lower components usually represent the inherent noise present in the data. Additionally, the use of a linear function in this study is mainly to make a simple model from a limited lamb wave data. The non-linear function would require considerable amount of data to estimate the parameters and introduces uncertainty during the model estimation phase.

## 3. Materials and Methods

**Figure 2:**
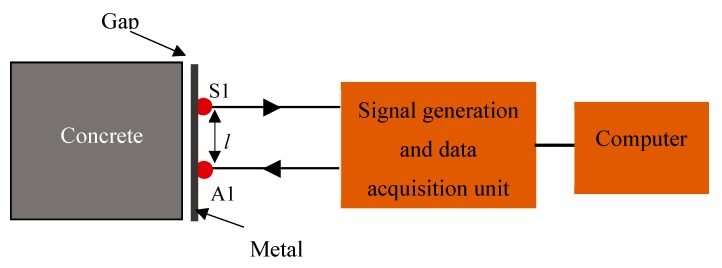
Schematic of experimental setup with two piezoelectric transducers: actuator (A1) and sensor (S1).

The instrumentation system consisted of two piezoelectric transducers, signal generation and data acquisition unit and a computer as shown in Figure 2. One transducer was used as an actuator (A1) and another as a sensor (S1). A circular piezoelectric transducer of diameter 6.35 mm was used. However, transducers of different shapes and sizes can also be used with the proposed method. The resonant frequency of the transducer in a radial mode was 300 kHz. The piezoelectric transducers were positioned 50 mm from each other and attached to the outer side of the metal plate of the composite structure. To find the optimal distance between the transducers, a range of distances from 25 to 200 mm were tested. The signal with the highest amplitude was determined at 50 mm. The piezoelectric transducers were connected to a signal generation and data acquisition unit from Acellent Technologies, which was in turn connected to a computer. A computer was used as a control centre that sent the command for signal generation and also acquired data from the transducers. A sinusoidal tone burst signal with 5 peaks and a peak-to-peak amplitude of 8 V was used as an excitation signal. The 5-peak burst signal was chosen because of its good dispersion characteristics and its sensitivity to structural flaws. Frequency sweeps from 100 to 500 kHz were performed at a step size of 100 kHz to determine the optimal measurement frequency and a frequency of 300 kHz was chosen due to the high amplitude of the received signal at this frequency. The signal generated by the actuator propagated along the metal plate of the composite structure and was detected by the sensor. The sampling rate was chosen to be 48 MS/s for data acquisition and a 40 dB gain was applied to the received signal.

A reference signal was taken where the metal was directly touching the concrete block without any gap. Then, signals were obtained at gap values of 0.1, 0.2, 0.3 and 0.4 mm. Thin paper sheets were used to create desired spacing between metal and concrete. The gaps were measured using a Vernier calliper. Multiple measurements were taken at the reference position and at each gap, which was then averaged and the standard deviation was calculated. The PLSR regression was carried out on the raw signal to extract features and determine a relationship between the signal and the damage (gap) size.

**Figure 3:**
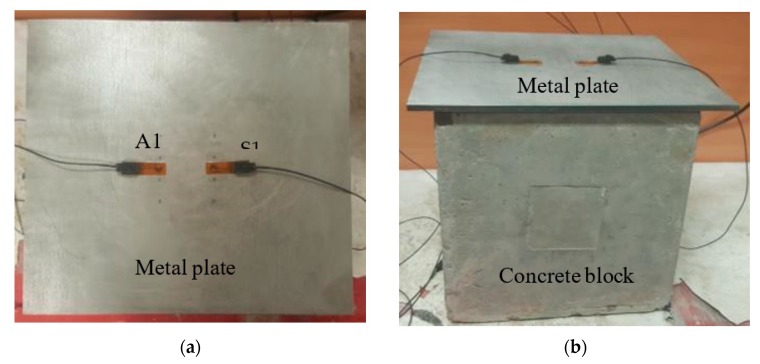
Photographic image of the experimental setup: (**a**) top view and (**b**) side view.

A photographic image of the experimental setup to detect the gap between the metal plate and concrete block is shown in Figure 3. A 250-mm concrete cube was prepared by mixing cement, sand, coarse aggregates and water, with appropriate proportions, and placed inside the mould for casting. The ratio of cement, sand and coarse aggregates was 1:2:3, respectively. Two different metal plates: 600 × 450 × 1.6 mm aluminium plate and 300 × 270 × 6 mm steel plate were placed on concrete at different stages of curing (from fresh concrete till 28 days) to form a composite structure. The thickness of the steel plate was close to the one used in structures such as CFSTs [45]. Although the main target specimen was a steel-concrete composite structure, the technique was first tested with a thinner aluminium plate for the comparative analysis.

## 4. Results and Discussion

**Figure 4:**
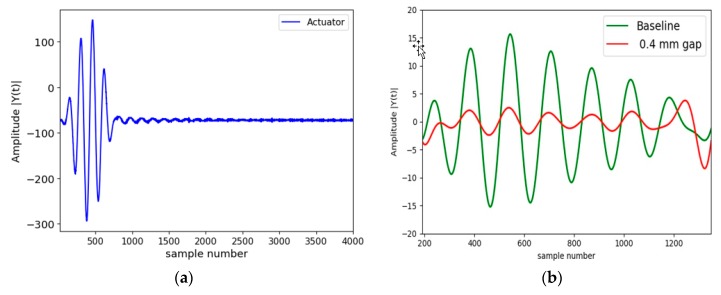
(**a**) Actuation signal and (**b**) sensor signal in steel-concrete composite structure.

The five-tone burst actuation signal is shown in Figure 4a, while the received signal at no gap (baseline) and at 0.4 mm gap (damage) in a 28-day old concrete is shown in Figure 4b. The signals in Figure 4b are the actual signals received by the piezoelectric sensors. The measurements were taken at different hours of concrete setting from the stage of casting until 28 days. PLSR was carried out on the received signal to get the gap prediction at different stages of concrete curing.

**Figure 5:**
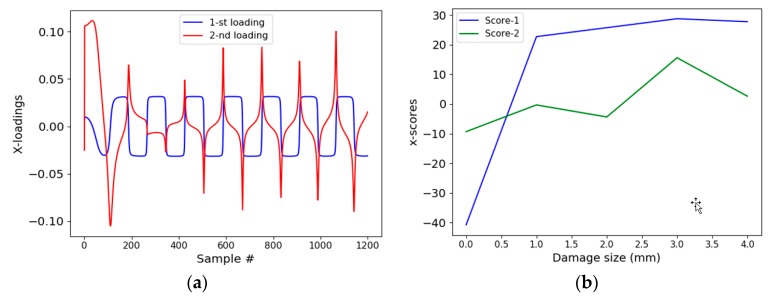
(**a**) 1st and 2nd X-loading vector estimated using partial least squared regression (PLSR); (**b**) 1st and 2nd X-score estimated after convoluting x-loading score and signal.

Figure 5 shows the first (1st) and the second (2nd) X-loading vector and X-scores estimated using the training data generated from the signal obtained before and after the damage. It can be seen from Figure 5a that the 1st loading vector resembles a step function. The convolution between the 1st loading vector and the actual signal results in the x-scores (Score-1) whose values can be seen in Figure 5b. The 2nd loading vector resembles more like a saw-tooth wave, the convolution of which, with the actual signal, produces Score-2 (reference Figure 5b). The 1st loading vector captures most of the information present in the signal, as the score magnitude for damage is 300% more than a score of an undamaged case.

**Figure 6:**
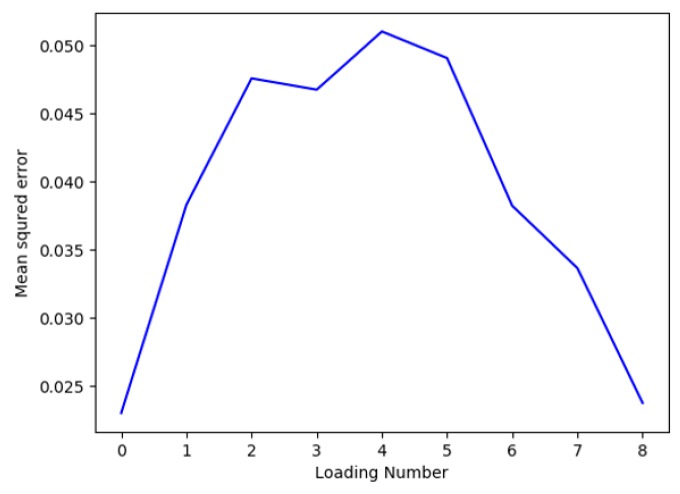
Mean squared error measured using five-fold cross-validation.

A five-fold cross-validation was performed to measure the mean squared error of PLSR with different numbers of loading vectors. It can be seen from Figure 6 that a least mean squared error of 0.0022 was obtained using the 1st loading vector. This is also consistent with our observation from Figure 5b that the 1st loading vector captures most of the variance in the data.

**Figure 7:**
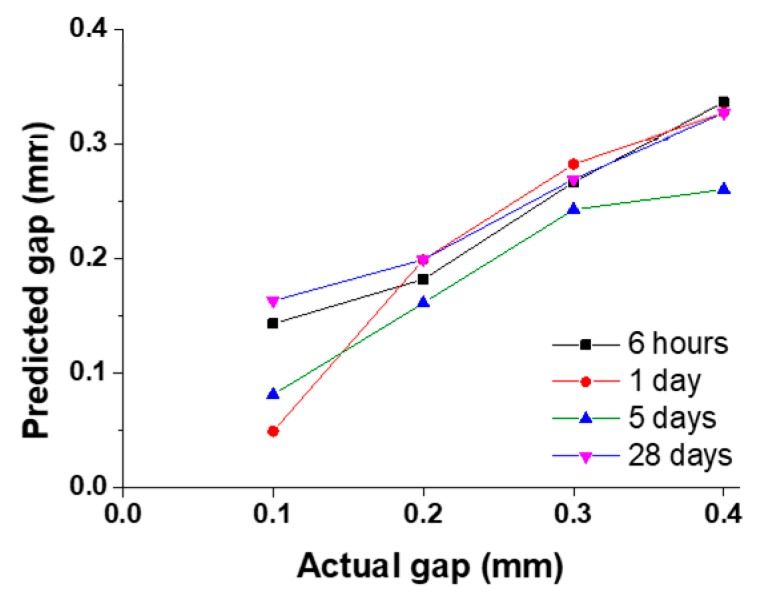
Predicted gap vs. actual gap between aluminium plate and concrete after 6 h, 24 h, 5 days and 28 days of concrete casting.

The gaps were predicted during different concrete curing stages using the aforementioned PLSR technique. The maximum standard deviation of the received signal at all gaps was determined to be 0.37. The higher standard deviation in some of the received signals was mainly due to the non-planarity of a metal plate. The predicted gaps vs. the actual gaps at 6 h, 24 h, 5 days and 28 days after concrete casting are shown in Figure 7. The figure demonstrates that the proposed methodology is able to measure gaps between the aluminium plate and concrete. The predicted gap values increased with the actual gap values. However, there were some irregularities in linearity of the predicted gap, which could be attributed to the vibration of the sample during testing due to the small thickness of the aluminium plate.

**Figure 8:**
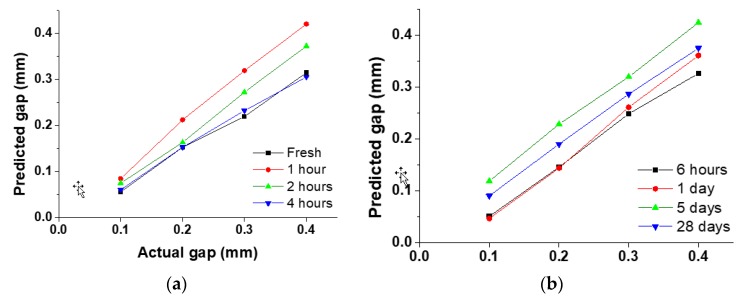
Predicted gap vs. actual gap between steel plate and concrete at (**a**) 0, 1, 2 and 4 h; and (**b**) 6 h, 1 day, 5 days and 28 days of concrete casting.

Next, the gaps were predicted in the composite structure composed of steel plate and concrete. The measurements were taken from fresh concrete (0 h) until 28 days at different stages of the concrete setting. The average standard deviation of the received signal at all gaps was determined to be 0.11. Figure 8a shows the predicted gap vs. actual gap at 0, 1, 2 and 4 h of concrete casting while Figure 8b shows these predictions after 6 h, 1 day, 5 days and 28 days. The gap predictions were more accurate and the linearity of the predicted gaps was higher compared to the previous specimen. This was mainly due to the thickness of the steel plate, which resulted in less vibration during the measurements. Further, the results show that predicted values were closer to the actual gap values as the concrete started to settle. To verify this, the correlation coefficient was determined between the predicted gap and the actual gap at different stages of the concrete setting. The correlation coefficient was determined using Equation (1).

(1) $$Correlation\;coefficient=\frac{n\sum^{\text{​}}xy-\left(\sum^{\text{​}}x\right)\left(\sum^{\text{​}}y\right)}{\sqrt{n\left(\sum^{\text{​}}x^{2}\right)-{\left(\sum^{\text{​}}x\right)}^{2}}-\sqrt{n\left(\sum^{\text{​}}y^{2}\right)-{\left(\sum^{\text{​}}y\right)}^{2}}}$$

where *n* is the number of points, *x* is the actual gap and *y* is the predicted gap at different stages of concrete setting.

**Figure 9:**
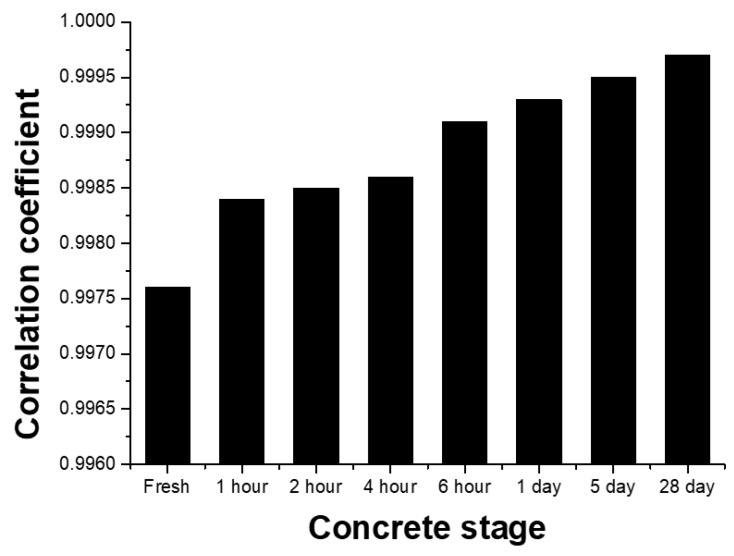
Correlation coefficient value between the actual and predicted gap in the steel-concrete composite specimen at different stages of concrete curing.

Figure 9 shows the comparison of the correlation coefficient between predicted and actual gaps at different stages of concrete setting, namely, 0 h, 1 h, 2 h, 4 h, 6 h, 1 day, 5 days and 28 days. The correlation coefficient between predicted and actual gaps at 0 h was found to be 0.9976 which increased to 0.9993 in one-day old concrete, which further increased to 0.9997 in 28-day old concrete (reference Figure 9). These results showed that the correlation coefficient was increasing linearly with time. It implies that a linear equation describes the relationship between the actual and predicted gap perfectly when the concrete is fully set. Thus, from this comparison, we can conclude that the accuracy of gap prediction is higher and more linear once the concrete is completely cured. This accuracy may be due to the high reflectivity of the set concrete resulting in the accurate prediction of the gaps. Further, the accuracy of the predicted gaps can be improved by using more lamb wave data, which will improve the training model for accurate prediction.

## 5. Conclusions

A PLSR-based method for the prediction of the gap in concrete-metal composite structures was proposed for the first time in this paper. This research expanded on previous principal component regression-based studies, wherein a more robust partial least squared regression-based method was utilised. This technique served a dual purpose of feature extraction and noise reduction. The developed technique utilised a single actuator and a single sensor attached to the external surface of the metal for non-invasive detection and prediction of gaps and debonding in composite structures. A gap width as small as 0.1 mm was predicted in concrete-based composite structures, with varying metal thickness. The detection and prediction of such minute gaps at an early stage enable one to take precautionary measures, preventing catastrophic failure of the structure. Moreover, the proposed technique can be utilised to detect debonding and delamination in diverse composite materials.
